# Assessment of Facial Asymmetry in Slovak Patients with Positional Deformity

**DOI:** 10.3390/children11121431

**Published:** 2024-11-26

**Authors:** Lenka Matejáková, František Horn, Petra Slaná, Andrej Plž, Maryam Zarinshad, Eva Štefánková

**Affiliations:** 1 Faculty of Medicine, Comenius University, 813 72 Bratislava, Slovakia; frantisek.horn@nudch.eu (F.H.); zarinshad1@uniba.sk (M.Z.); eva.stefankova@nudch.eu (E.Š.); 2 Department of Pediatric Neurosurgery, National Institute of Children’s Diseases, 833 40 Bratislava, Slovakia; 3 Department of Anthropology, Faculty of Natural Sciences, Comenius University, 841 04 Bratislava, Slovakia; slana13@uniba.sk; 4 PROTEA, s.r.o., 821 01 Bratislava, Slovakia; plz@protea.sk; 5 Department of Pediatric Surgery, National Institute of Children’s Diseases, 833 40 Bratislava, Slovakia

**Keywords:** positional deformity, facial asymmetry, 3D scanning, anthropometry

## Abstract

**Objectives:** Positional deformity (PD), also known as deformational plagiocephaly or non-synostosis, is a primary cause of abnormal head shape and asymmetry in infants. The most common type, occipital plagiocephaly, leads to flattening of one side of the back of the head or the entire head (positional brachycephaly). PD results from external forces on the growing skull, often due to childbirth and improper positioning during sleep. The incidence is approximately 1 in 300 births, with prevalence peaking between the seventh week and four months of age. Our objective was to monitor craniofacial parameters in patients with positional deformity (PD), to evaluate cranial asymmetry and PD severity, and to determine the relationship between craniofacial asymmetry and PD severity. **Methods:** This study included patients from the Department of Pediatric Neurosurgery at the National Institute of Children’s Diseases and the Faculty of Medicine, Comenius University in Bratislava, Slovakia. Craniofacial parameters on the right and left sides of the face were examined. **Results:** Significant differences were found in the upper and middle thirds of the face, indicating a strong association between positional deformity and facial asymmetry. **Conclusions:** the findings contribute new insights into craniofacial anthropometry and neurosurgery, enhancing the diagnosis of positional deformity in Slovakian patients.

## 1. Introduction

Positional deformity (PD), also referred to as deformational plagiocephaly or non-synostotic plagiocephaly, is one of the primary causes of abnormal head shape and cranial asymmetry in infants. It is a craniofacial anomaly characterized by an asymmetrical skull shape resulting from prolonged external pressure on a baby’s head, often during sleep or other static activities. The prevalence of PD has notably increased since the introduction of the “Back to Sleep” campaign, which encourages infants to sleep on their backs to reduce the risk of sudden infant death syndrome (SIDS) [1,2,3].

The most frequent type of PD is occipital plagiocephaly, which manifests as flattening on one side of the back of the head. In some cases, flattening affects the entire back of the head, a condition known as positional brachycephaly. Positional deformities can arise due to external forces acting on the developing skull, either prenatally or in early infancy, and are influenced by various biological and environmental factors [4,5].

The etiology of PD is multifactorial and includes prenatal risk factors such as in utero positioning and multiple pregnancies, perinatal factors like assisted or premature births, and postnatal influences, most notably prolonged supine sleeping positions or restricted head movement associated with torticollis. These combined factors underscore the complexity of PD’s origins and progression [5].

The clinical features of PD vary by severity and may include visible cranial asymmetry, uneven ear alignment, facial asymmetry, and in severe cases, deformation affecting the temporomandibular joint. Although rare, PD has also been linked to neuromotor delays and cognitive impairments, though aesthetic concerns remain the most commonly reported issue. Diagnosis primarily relies on clinical evaluation, where the infant’s head shape and asymmetry are assessed by a physician. Advanced diagnostic tools such as anthropometry offer precise cranial measurements, while 3D scanning enables detailed analysis of the skull’s anatomical structure, aiding in the accurate classification of deformities.

Treatment for PD is tailored to the severity of the condition and the patient’s individual needs. Options range from non-invasive approaches like physical therapy and repositioning strategies to cranial remodeling orthoses and helmets. Surgical intervention is rarely required and is typically reserved for severe or refractory cases [6,7,8].

The incidence of positional deformities is estimated at approximately 1 in 300 births, with prevalence peaking between the sixth week and four months of life (16.0–46.0%) before stabilizing at around 3.3% by two years of age. This increase in reported cases is partly attributed to heightened awareness among healthcare professionals and parents about positional deformities [2].

This study aims to evaluate craniofacial asymmetry in Slovak infants with positional deformities, with a particular focus on deformational plagiocephaly. To our knowledge, this is the first investigation of its kind conducted in Slovakia, providing essential baseline data on the prevalence and severity of facial asymmetry related to positional deformities within this population. These findings will contribute to a deeper understanding of the condition and support the development of evidence-based clinical practices for early detection and intervention in Slovakia.

## 2. Materials and Methods

### 2.1. Patients

The monitored group consisted of 55 individuals aged 2.43–11.20 months (average age 5.99 ± 2.07 months), with positional deformities, coming from different parts of Slovakia. Patients with possible craniosynostosis or other types of craniofacial deformities were excluded. Of the total number of individuals, there were 18 girls with an average age of 5.59 ± 1.90 months and 37 boys with an average age of 6.18 ± 2.15 months. Only the initial examinations of patients were considered (in cases where a patient underwent multiple anthropometric evaluations), and the cohort included only patients who had not yet undergone any correction for the deformity. Data collection took place at the National Institute of Children’s Diseases at the Department of Pediatric Surgery in Bratislava. The group consisted of patients suffering from positional deformity, who were subsequently divided into three categories according to the severity of the deformity (Table 1). Patients who, according to Table 1, fell into the “no deformity” or “minor deformity” category were classified under “minor deformity”, patients in the “moderate simple” and “moderate combined” categories were classified under “moderate deformity”, and patients in the “severe simple” and “severe combined” categories were classified under “severe deformity”. This project was approved by the Ethics Committee of the National Institute of Children’s Diseases, project number EK12/2/2022. Informed consent has been obtained from all relevant persons (such as the parent or legal guardian) included in this study.

### 2.2. Three-Dimensional Scanning and Anthropometry

**Figure 1:**
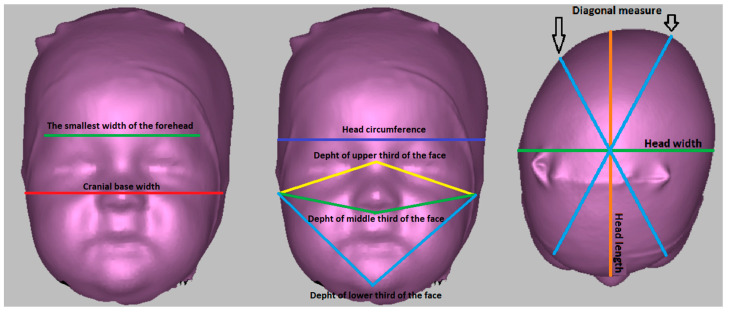
Anthropometric measurements performed on a 3D scanner.

The 3D scanning technique using the Artec Eva (Artec 3D, Findel Senningerberg, Luxembourg) 3D scanner was used to obtain the data. This handheld scanner is user-friendly and utilizes safe structured light scanning technology to create textured and accurate 3D models of various objects, including the human body. After positioning the scan in the Frankfurt horizontal plane, the dimensions were measured (Figure 1).

Head circumference—circumference measured from the glabella point parallel to the Frankfurt horizontal plane.

Head length—direct measurement of the distance between the glabella point and the intersection between the head circumference and the central plane perpendicular to the Frankfurt horizontal plane.

Head width—the largest head width measured perpendicular to the medial plane and Frankfurt horizontal plane.

Diagonal measure—a diagonal inclined on both sides by 30° to the mid-sagittal plane, perpendicular to the Frankfurt horizontal plane.

The smallest width of the forehead—the direct distance between the intersections of the right diagonal measure with the head circumference and the left diagonal measure with the head circumference.

Cranial base width—the direct distance between the tragion points.

Depth of the upper third of the face—direct distance between the tragion and nasion points.

Depth of the middle third of the face—direct distance between the tragion and subnasale points.

Depth of the lower third of the face—the direct distance of the tragion and pogonion points.

Index cephalicus (IC)—defined as the ratio of head width to length multiplied by 100 (%).

IC = head width/head length × 100(1)

Cranial vault asymmetry index (CVAI)—defined as the ratio of the difference between the smaller (Dg1) and larger (Dg2) diagonal dimensions and the smaller diagonal dimension multiplied by 100 (%).

CVAI = Dg1 − Dg2/Dg2 × 100(2)

### 2.3. Statistical Analysis

The data obtained were processed in Microsoft Excel (Microsoft Corporation, Redmond, WA, USA) and IBM SPSS version 25.0 (International Business Machines Corp., Armonk, NY, USA). The Mann–Whitney U test was used to compare the differences in the values of the monitored dimensions between boys and girls. A paired Wilcoxon *t*-test was used to monitor facial asymmetry. Kruskal–Wallis test was used to determine the relationships between the degree of deformity severity and facial asymmetry and also between deformity symmetry and facial asymmetry.

## 3. Results

In the observed group, the majority of patients were classified as having severe deformities, accounting for 67.2% of the cohort. This was followed by patients with moderate deformities, who represented 27.3% of the group, while the smallest proportion of patients, 5.5%, fell into the category of mild deformities. These findings highlight that a significant portion of the monitored group exhibited severe cranial deformities, indicating the predominance of more advanced cases in this cohort.

When further dividing the patients based on the symmetry of their deformities, the data revealed 24 cases of symmetrical deformities (43.6%), 1 case of an asymmetrical deformity (1.8%), and 30 cases of combined deformities (54.6%). This distribution underscores that symmetrical deformities are more frequent than asymmetrical ones within this group. Notably, the most common presentation involved a combination of positional plagiocephaly and positional brachycephaly. These findings suggest that combined deformities are a prevailing pattern in children with positional cranial deformities.

The characteristics of the boys and girls in the cohort, with regard to craniofacial parameters and indices, are summarized in Table 2. Statistically significant differences between boys and girls were observed for certain craniofacial measurements. Specifically, boys exhibited significantly higher mean values for head circumference (*p* = 0.022) and the right diagonal dimension (*p* = 0.027). Additionally, boys had higher measurements for the upper third (*p* = 0.007), middle third (*p* = 0.001), and lower third (*p* = 0.002) of the face on the right side, as well as the corresponding measurements on the left side of the face (*p* = 0.010, *p* = 0.002, *p* = 0.005). These intersex differences are consistent with the general observation that male children tend to have larger craniofacial dimensions, which may reflect their overall larger body size at birth. Conversely, parameters such as head length, head width, forehead width, and cranial base width did not show statistically significant intersex differences. This lack of significant differences may be due to the influence of the cranial deformities themselves, which could obscure inherent differences between sexes in these dimensions. After applying the Bonferroni correction to account for multiple comparisons, the craniofacial parameters that remained significantly different between boys and girls were T-SN right and T-PG right, indicating consistent and robust intersexual differences for these specific measurements. These findings emphasize the importance of these parameters as reliable indicators of intersex craniofacial variation in the presence of positional deformities.

In the monitored group of patients, bilateral differences in craniofacial parameters were analyzed to assess facial asymmetry, as shown in Table 3. Statistically significant differences were observed in the depth of the upper third of the face (*p* = 0.045) and the depth of the middle third of the face (*p* = 0.016). These findings suggest that positional deformities can impact the symmetry of these facial regions, potentially leading to subtle but measurable differences in craniofacial morphology.

In contrast, no statistically significant bilateral differences were found in the lower third of the face, indicating that this region does not exhibit asymmetry in patients with positional deformities. Specifically, the mandible area appears to remain unaffected, preserving its symmetry even in the presence of cranial deformation. This finding supports the notion that lower facial structures are less influenced by external pressures that cause asymmetry in other regions of the face, such as the upper and middle thirds.

It is important to note that the observed differences in asymmetry, particularly in the upper and middle thirds of the face, are extremely small—on the scale of millimeters or even tenths of a millimeter. As a result, these differences are often imperceptible to the naked eye and may not have a noticeable impact on the overall appearance of the face. However, their detection through precise measurement methods highlights the value of detailed anthropometric analysis and 3D imaging in identifying subtle variations that would otherwise go unnoticed.

These findings contribute to our understanding of how positional deformities selectively affect different regions of the face, with certain areas, such as the upper and middle thirds, being more prone to asymmetry, while others, like the lower third, remain largely unaffected. This distinction could have implications for both diagnosis and targeted treatment strategies, particularly in addressing aesthetic and functional concerns associated with craniofacial asymmetry.

When analyzing facial asymmetry in relation to the symmetry and severity of the deformity, no statistically significant associations were found. This suggests that in the monitored group, the degree of facial asymmetry does not vary based on whether the deformity is classified as symmetrical or asymmetrical, nor does it correlate with the severity of the deformity.

These findings indicate that facial asymmetry in positional cranial deformities may arise independently of these two factors. It is possible that other variables, such as the specific location and type of external pressure on the skull or underlying individual anatomical differences, play a more significant role in the development of facial asymmetry than the overall symmetry or severity classification of the deformity.

In summary, our findings suggest that facial asymmetry in children with positional deformities is not directly influenced by the severity or symmetry of the condition, emphasizing the need for further research to identify the factors contributing to craniofacial asymmetry. However, it is important to note that the majority of patients in our cohort had severe deformities, which could influence these findings by skewing the distribution of asymmetry-related outcomes. A more balanced representation of patients across all severity levels may provide additional insights and a more comprehensive understanding of the relationship between deformity characteristics and facial asymmetry.

These results may help refine diagnostic and treatment strategies to better address the nuances of positional deformities and their impact on facial features, particularly in cases with a broader range of deformity severity.

## 4. Discussion

Regarding the facial asymmetry of the patients in the monitored group, we found, on average, higher values of facial asymmetry parameters on the left side of the face, while these values were lower on the right. When comparing the craniofacial parameters of facial asymmetry of the right and left sides of the face based on 3D scans, we found statistically significant differences between the right and left sides of the upper and middle third of the face. Nakamori et al. [10], in their study, focused on positional deformities in preterm infants as well as in full-term infants, observing them over three assessments. In this study, the authors used the same scanner model and procedures to calculate CVAI and CI as in our study. However, when measuring asymmetry in the upper third of the face, they used the sellion point instead of the nasion point, which is very similar. The sellion point is located in the deepest part of the nasal root. In full-term patients, they recorded only mild occipital asymmetry throughout this study, with the worst results during the second assessment; however, by the third assessment, statistically significant improvements were observed. Brachycephaly was recorded in approximately 22% of patients at the final assessment. In our study, the prevalence of brachycephaly was almost 100%; however, we observed patients who had not yet undergone any treatment, and only initial examinations were included in our sample, i.e., before any intervention. The average prevalence of brachycephaly in Slovakia is currently unknown; however, a trend of debrachycephalization has been observed [11]. Kato et al. [12] also examined asymmetry in the occipital and frontal regions, comparing two 3D techniques—the anterior symmetry ratio (ASR) and the posterior symmetry ratio (PSR), as well as their combination. Similarly to the previous study, the same scanner model and procedures were used to calculate CVAI and CI, and the authors also used the sellion point when examining asymmetry in the upper third of the face. However, in this study, the authors additionally divided the cranium into four quadrants, which were later used to calculate the ASR and PSR. Using ASR, they found 97.8% of patients with mild deformity and 1.3% with severe deformity. With PSR, 82.6% of patients showed mild asymmetry, while 17.4% exhibited severe asymmetry. When using both techniques, moderate asymmetry (when asymmetry was detected by only one method) was observed in 81.5% of patients, and severe asymmetry (detected by both methods) was noted in 18.5% of patients. Our results correspond with the results of the study by Baratta et al. [13], who found facial asymmetry in patients with torticollis based on 3D stereophotogrammetry, with both the cameras and the patient positioned in a standardized setup. To calculate asymmetry in the three thirds of the face, the authors used the root-mean-square deviation (RMSD), which measures the difference between the true face and the mirrored face. They showed a greater degree of facial asymmetry mainly in the upper and middle third of the face but did not detect significant asymmetry in the lower third of the face. Kreutz et al. [14], in their study, also used 3D stereophotogrammetry with a special highchair to prevent movements. They monitored CVAI and CI, along with asymmetry in the three thirds of the face, using the same methods as in our study (measuring distances T-N, T-SN, and T-PG on both sides of the face), while they observed the correction of facial asymmetry after remodeling helmet therapy in infants with an average age of five months, demonstrating the presence of asymmetry in the middle and lower third of the face in patients with positional plagiocephaly before correction. The results of the study by Naros et al. [15] suggest that positional plagiocephaly in most cases leads to significant facial asymmetry in all directions and areas of the face. Helmet therapy resulted in a significant reduction in intra-individual facial asymmetry. However, they did not observe any correlation between the cranial vault asymmetry index and facial asymmetry. In this study, 3D stereophotogrammetry was also used. Baumler et al. [16] observed asymmetry in positional plagiocephalies based on 3D CT scans of children with PD. The monitored children were divided into two groups: 51 with frontal plagiocephaly and 19 with occipital plagiocephaly. The jaw was symmetrical in the case of occipital plagiocephaly, while it showed signs of asymmetry in frontal plagiocephaly. The asymmetry of the jaw was variable, but in most cases, it tended to compensate for the asymmetry of the skull base, which was particularly pronounced in frontal plagiocephaly. In Miyabayashi et al.’s [17] study, they measured IC and CVAI in patients from Japan. In contrast to our study, they divided patients according to CVAI into categories of mild, moderate, severe, and very severe deformity. They recorded 37.9% of patients with mild deformity, 20.3% of patients with moderate deformity, 5.9% severe, and 0.7% very severe. This means that the majority of patients in this study fell into the category of mild deformity, in contrast to our results, where we recorded a majority of patients with severe deformity. They also used the same 3D scanner as in our study; however, instead of the nasion point, they used the sellion point. In the study by Akutsu et al. [18], they followed patients with positional deformity of different ages, while patients of a similar age to our group were included in the first group (2–23 months of age). Mild deformities were noted in more than half of the patients, about a third of the patients suffered from moderate deformities, and 9.7% suffered from severe deformities. In both mentioned studies, the individual categories of deformity severity were divided differently than in our study. Akutsu et al. [18] also followed positional deformity on CT images rather than 3D scans.

By observing the craniofacial parameters of facial asymmetry on the right and left sides of the face, we found statistically significant differences in the parameters of the depth of the upper and middle third of the face. Since the jaw is connected to the rest of the skull by articulation and is not affected to such an extent by the deformation caused by positional plagiocephaly, we did not observe statistically significant differences in the depth of the lower third of the face. The results confirm that positional plagiocephaly in most cases is also related to the presence of facial asymmetry. One potential limitation of our study is the influence of selection bias, as our sample consists exclusively of patients referred for medical evaluation. It is likely that individuals with milder forms of plagiocephaly were underrepresented, as they may not have sought assessment or intervention. Consequently, our findings may reflect an overrepresentation of more severe cases. While we aimed to mitigate this by including all patients referred during the study period, the generalizability of our results to the broader population may be limited. Future research should consider community-based studies to capture a more comprehensive spectrum of plagiocephaly severity and reduce referral-related bias.

## 5. Conclusions

This original study, the first of its kind in Slovakia, highlights the significant impact of positional deformity (PD) on craniofacial asymmetry in infants. By thoroughly examining craniofacial parameters in patients using new technology in clinical anthropology, we have identified notable differences in the upper and middle thirds of the face associated with PD. These findings underscore the importance of early detection and intervention for positional deformities to mitigate long-term craniofacial asymmetry. Healthcare professionals, particularly those in pediatrics and neurosurgery, should be aware of the prevalence and characteristics of PD. Early diagnosis and appropriate management, including education on proper infant positioning and potential therapeutic interventions, are crucial. Increased awareness and understanding of PD among caregivers can lead to better prevention strategies, reducing the incidence and severity of craniofacial asymmetries. This study contributes valuable insights to the fields of craniofacial anthropometry and pediatric neurosurgery, providing a foundation for further research and improved clinical practices in diagnosing and treating positional deformity. By advancing our knowledge of PD through innovative technological methods, we can enhance patient outcomes and quality of life for affected infants and their families.

## Figures and Tables

**Table 1:** Distribution of severity and type of deformity based on index cephalicus and cranial vault asymmetry index according to Lipina et al. [9]. Combined categories represent cases where both IC and CVAI indicate similar severity levels, while “simple” refers to deformities predominantly affecting one measure.

Deformity Based on IC	Deformity Based on CVAI			
	No Deformity < 3.5%	Minor 3.6–9.0%	Moderate 9.1–13.5%	Severe > 13.5%
Severe > 93.0%	Severe simple	Severe combined	Severe combined	Severe combined
Moderate 88.1–93.0%	Moderate simple	Severe combined	Severe combined	Severe combined
Minor 83.1–88.0%	Minor simple	Moderate combined	Severe combined	Severe combined
No deformity < 83.0%	No deformity	Minor simple	Moderate simple	Severe simple

IC—index cephalicus; CVAI—cranial vault asymmetry index.

**Table 2:** Intersexual differences in craniofacial parameters and indices in the monitored group.

Cranial Parameters and Indices	Girls			Boys			*p*	*p* Adj.
	N	Mean	SD	N	Mean	SD		
HC (cm)	18	42.68	2.10	37	43.69	2.16	0.022 *	0.396
HL (cm)	18	14.10	0.88	37	14.32	0.86	0.076	1.000
HW (cm)	18	12.81	0.81	37	13.05	0.74	0.158	1.000
FW (cm)	18	11.94	0.67	37	7.33	0.80	0.232	1.000
CBW (cm)	16	10.61	0.69	37	11.94	0.67	0.146	0.486
DG right (cm)	18	13.67	0.71	37	14.35	0.82	0.027 *	1.000
DG left (cm)	18	4.70	3.31	37	14.15	0.76	0.130	1.000
T-N right (cm)	16	8.46	0.52	37	9.17	0.56	0.007 **	0.126
T-N left (cm)	16	8.87	0.31	37	9.24	0.57	0.010 *	0.180
T-SN right (cm)	16	8.42	0.64	37	9.32	0.52	0.001 ***	0.018 *
T-SN left (cm)	16	8.95	0.33	37	9.43	0.56	0.002 **	0.036 *
T-PG right (cm)	15	8.74	0.64	34	9.55	0.61	0.002 **	0.036 *
T-PG left (cm)	15	9.10	0.38	34	9.61	0.61	0.005 **	0.090
IC (%)	18	91.00	5.42	37	91.25	4.54	0.486	1.000
CVAI (%)	18	4.80	3.00	37	4.69	3.31	0.690	1.000
T-N diff (cm)	16	0.20	0.19	37	0.23	0.19	0.568	1.000
T-SN diff (cm)	16	0.18	0.19	37	0.22	0.20	0.516	1.000
T-PG diff (cm)	15	0.16	0.13	34	0.21	0.17	0.455	1.000

N—number of individuals; SD—standard deviation; *p*—achieved level of significance; *p* Adj.—*p*-value adjusted with Bonferroni correction; HC—head circumference; HL—head length; HW—head width; FW—forehead width; CBW—cranial base width; CVAI—cranial vault asymmetry index; T-N—depth of the upper third of the face; T-SN—depth of middle third of the face; T-PG—depth of lower third of the face; IC—index cephalicus; diff—difference, * —*p* ≤ 0.05, ** —*p* ≤ 0.01, *** —*p* ≤ 0.001

**Table 3:** Comparison of craniofacial parameters of facial asymmetry of the right and left sides of the face.

Cranial Parameters and Indices	Right Side			Left Side			*p*
	N	Mean	SD	N	Mean	SD	
T-N (cm)	53	9.04	0.54	53	9.11	0.56	0.045 *
T-SN (cm)	53	9.16	0.52	53	9.27	0.57	0.016 *
T-PG (cm)	49	9.38	0.61	59	9.45	0.61	0.090

N—number of individuals; SD—standard deviation; *p*—achieved level of significance; T-N—depth of the upper third of the face; T-SN—depth of the middle third of the face; T-PG—depth of the lower third of the face, * —*p* ≤ 0.05.

## Data Availability

The data presented in this study are available on request from the corresponding author due to privacy and ethical restriction, as they contain sensitive information.

## References

[1] Linz C., Kunz F., Böhm H., Schweitzer T. (2017). Positional Skull Deformities. Dtsch. Arztebl..

[2] Vallamkonda N., Maria A. (2017). Positional Plagiocephaly. Int. J. Adv. Sci. Res. Manag..

[3] Jung B.K., Yun I.S. (2020). Diagnosis and treatment of positional plagiocephaly. Arch. Craniofac. Surg..

[4] Kabbani H., Raghuveer T.S. (2004). Craniosynostosis. Am. Fam. Physician.

[5] Mawji A., Vollman A.R., Fung T., Hatfield J.M., Mcneil D.A., Sauvé R. (2014). Risk factors for positional plagiocephaly and appropriate time frames for prevention messaging. Paediatr. Child. Health.

[6] Inchingolo A.D., Inchingolo A.M., Piras F., Malcangi G., Patano A., Di Pede C., Netti A., Ciocia A.M., Corriero A., Semjonova A. (2022). Systematic Review of Positional Plagiocephaly Prevention Methods for Patients in Development. Appl. Sci..

[7] Unnithan A.K.A., De Jesus O. (2024). Plagiocephaly. StatPearls.

[8] Gandolfi F.A., Villani D., Meraviglia M.V. (2014). Definition and Classification. Positional Plagiocephaly.

[9] Lipina R., Rosický J., Golová Š. (2012). Treatment of positional plagiocephaly using a cranial molding orthosis. Padiatr. Praxi.

[10] Nakanomori A., Miyabayashi H., Tanaka Y., Maedomari T., Mukai C., Saito K., Okahashi A., Nagano N., Morioka I. (2023). Chan-ges in Cranial Shape and Developmental Quotient at 6 Months of Age in Preterm Infants. Children.

[11] Fuchsová M., Fagalová L., Beňuš R., Neščáková E., Bodoriková S. (2014). Trends in the growth of head and facial dimensions of children from 6 to 15 years of age in Slovakia. Slov. Antropol..

[12] Kato R., Nagano N., Hashimoto S., Saito K., Miyabayashi H., Noto T., Morioka I. (2022). Three-Dimensional versus Two-Dimensional Evaluations of Cranial Asymmetry in Deformational Plagiocephaly Using a Three-Dimensional Scanner. Children.

[13] Baratta V.M., Linden O.E., Byrne M.E., Sullivan S.R., Taylor H.O. (2022). A Quantitative Analysis of Facial Asymmetry in Torticollis Using 3-Dimensional Photogrammetry. Cleft Palate Craniofacial J..

[14] Kreutz M., Fitze B., Blecher C., Marcello A., Simon R., Cremer R., Zeilhofer H.F., Kunz C., Mayr J. (2018). Facial asymmetry correc-tion with moulded helmet therapy in infants with deformational skull base plagiocephaly. J. Craniomaxillofac. Surg..

[15] Naros A., Wolf J.A., Krimmel M., Kluba S. (2021). Three-Dimensional Quantification of Facial Asymmetry in Children with Positional Cranial Deformity. Plast. Reconstr. Surg..

[16] Baumler C., Lebouocq N., Captier G. (2007). Mandibular asymmetry in plagiocephaly without synostosis. Rev. Stomatol. Chir. Max-illofac..

[17] Miyabayashi H., Nagano N., Kato R., Noto T., Hashimoto S., Saito K., Morioka I. (2022). Reference Values for Cranial Morphology Based on Three-dimensional Scan Analysis in 1-month-old Healthy Infants in Japan. Neurol. Med. Chir..

[18] Akutsu N., Koyama J., Kawamura A., Sasayama T. (2024). Prevalence and Severity of Positional Posterior Plagiocephaly and Position-al Posterior Brachycephaly in Children and Adolescents in Japan. Neurol. Med. Chir..

